# 

**Published:** 1891-08

**Authors:** 


*Entered at the Post Office at Austin, Texas, as Second-Class Mail Matter.*

VOL. VII. AUGUST, 1891. No. 2.

### DANIEL'S MEDICAL JOURNAL

Subscription, $2.00 a Year, in Advance.—Single Copies, Twenty-five Cents.

F. E. DANIEL, M. D., Editor and Publisher, AUSTIN, TEXAS.

